# Dual hemostatic defects in Glanzmann thrombasthenia and von Willebrand disease: functional characterization of ITGA2B and dominant-negative von Willebrand factor variants

**DOI:** 10.1016/j.rpth.2026.106851

**Published:** 2026-07-14

**Authors:** Yang Zhang, Takahiro Shima, Ikumi Yamanaka, Saori Ueda, Aoi Nishida, Shinya Ohga, Yosuke Morodomi, Akihiko Numata, Tetsuya Eto, Taisuke Kanaji, Takashi Okamura, Koji Kato, Koichi Akashi, Yuya Kunisaki, Sachiko Kanaji

**Affiliations:** 1 Department of Clinical Chemistry and Laboratory Medicine, Graduate School of Medical Sciences, Kyushu University, Fukuoka, Japan; 2 Department of Medicine and Biosystemic Science, Graduate School of Medical Sciences, Kyushu University, Fukuoka, Japan; 3 Division of Medical Oncology, Hematology and Infectious Disease, Department of Internal Medicine, Fukuoka University Faculty of Medicine, Fukuoka, Japan; 4 R&D Laboratory for Innovative Biotherapeutics Science, Graduate School of Pharmaceutical Sciences, Kyushu University, Fukuoka, Japan; 5 Department of Hematology, Hamanomachi Hospital, Fukuoka, Japan; 6 Department of Integrative Structural and Computational Biology, The Scripps Research Institute, La Jolla, California, USA; 7 Department of Hematology, St. Mary's Hospital, Kurume, Japan

Glanzmann thrombasthenia (GT) is a rare autosomal recessive bleeding disorder characterized by defective or absent platelet aggregation due to quantitative or qualitative abnormalities of the platelet integrin α_IIb_β_3_ complex [1]. The prevalence of GT is estimated at approximately 1 per million individuals, and affected patients typically present with bleeding diathesis from early childhood [2,3].

By contrast, von Willebrand disease (VWD) is the most common inherited bleeding disorder, caused by quantitative or qualitative abnormalities of von Willebrand factor (VWF), a key glycoprotein that mediates platelet adhesion and stabilizes factor (F)VIII [4]. The clinical manifestations of these disorders—epistaxis, gum bleeding, and menorrhagia—often overlap, which can obscure diagnosis when they coexist. Reports of concurrent GT and VWD are exceedingly rare, and the molecular mechanisms underlying such dual hemostatic defects have not been previously clarified in detail [5].

Here, we report a patient with GT who presented with persistent bleeding unresponsive to platelet transfusions and was subsequently diagnosed with concomitant VWD. Comprehensive genetic and biochemical analyses identified a homozygous *ITGA2B* frameshift mutation and a heterozygous *VWF* missense mutation that affect VWF secretion in a dominant-negative manner. Gastrointestinal bleeding caused by angiodysplasia is recognized in VWD but is not common in platelet disorders, including GT. Thus, this case suggests the importance of considering coexisting VWD when evaluating atypical bleeding manifestations in patients with platelet disorders.

A 53-year-old woman has a lifelong history of bleeding symptoms. Her family history revealed consanguinity between her parents. Subcutaneous bruising was first noted at 2 months of age, and recurrent epistaxis developed at age 3 years. At age 5 years, she was diagnosed with GT based on the absence of platelet aggregation responses. From adolescence, she experienced intermittent gastrointestinal bleeding, which was managed with platelet transfusions.

At age 20 years, she developed massive gastrointestinal bleeding refractory to platelet transfusion, prompting referral to our hospital. Laboratory evaluation revealed reduced plasma VWF antigen (23%) and VWF ristocetin cofactor activity (35%), leading to the diagnosis of concurrent VWD. At age 22 years, she experienced recurrent gastrointestinal bleeding that was difficult to control. Angiography demonstrated an increased number of dilated blood vessels, with contrast medium leakage from the ileum. Embolization failed to control the recurrent ileal bleed, necessitating segmental bowel resection. Intraoperative endoscopic examination identified multiple red spots with oozing blood throughout the ileum and jejunum. Histological evaluation demonstrated a marked increase in dilated blood vessels in the lamina propria mucosae and submucosa, findings consistent with angiodysplasia.

Repeated platelet transfusions led to the development of anti-human leukocyte antigen antibodies, necessitating human leukocyte antigen-matched platelet transfusions thereafter. For subsequent invasive procedures, she was managed with both human leukocyte antigen-matched platelet transfusions and plasma-derived VWF/FVIII concentrates.

She has been followed up regularly at a local hospital and was recently readmitted to our hospital for planned perioperative management of hemostasis in anticipation of oral surgery. Taking this opportunity, we obtained informed consent from the patient and conducted genetic and biochemical analyses to elucidate the detailed pathophysiology of bleeding resulting from the coexistence of GT and VWD.

On admission, her complete blood count and coagulation profile were within normal limits (white blood cell count: 4.18 × 10^9^/L; hemoglobin: 12.2 g/dL; platelet count: 231 × 10^9^/L; prothrombin time: 11.4 seconds; activated partial thromboplastin time: 30.9 seconds). Her platelet size (mean platelet volume: 11.3 fL) was within normal range (9.1-12.4). Her plasma VWF antigen was 41%, and VWF ristocetin cofactor activity was 24%. FVIII activity was normal (85%). It remains unclear whether the higher VWF antigen level than that at diagnosis >30 years ago reflects the well-recognized age-related increase in VWF, stress-related fluctuations, or differences in assay methods used at that time [6].

**Figure 1:**
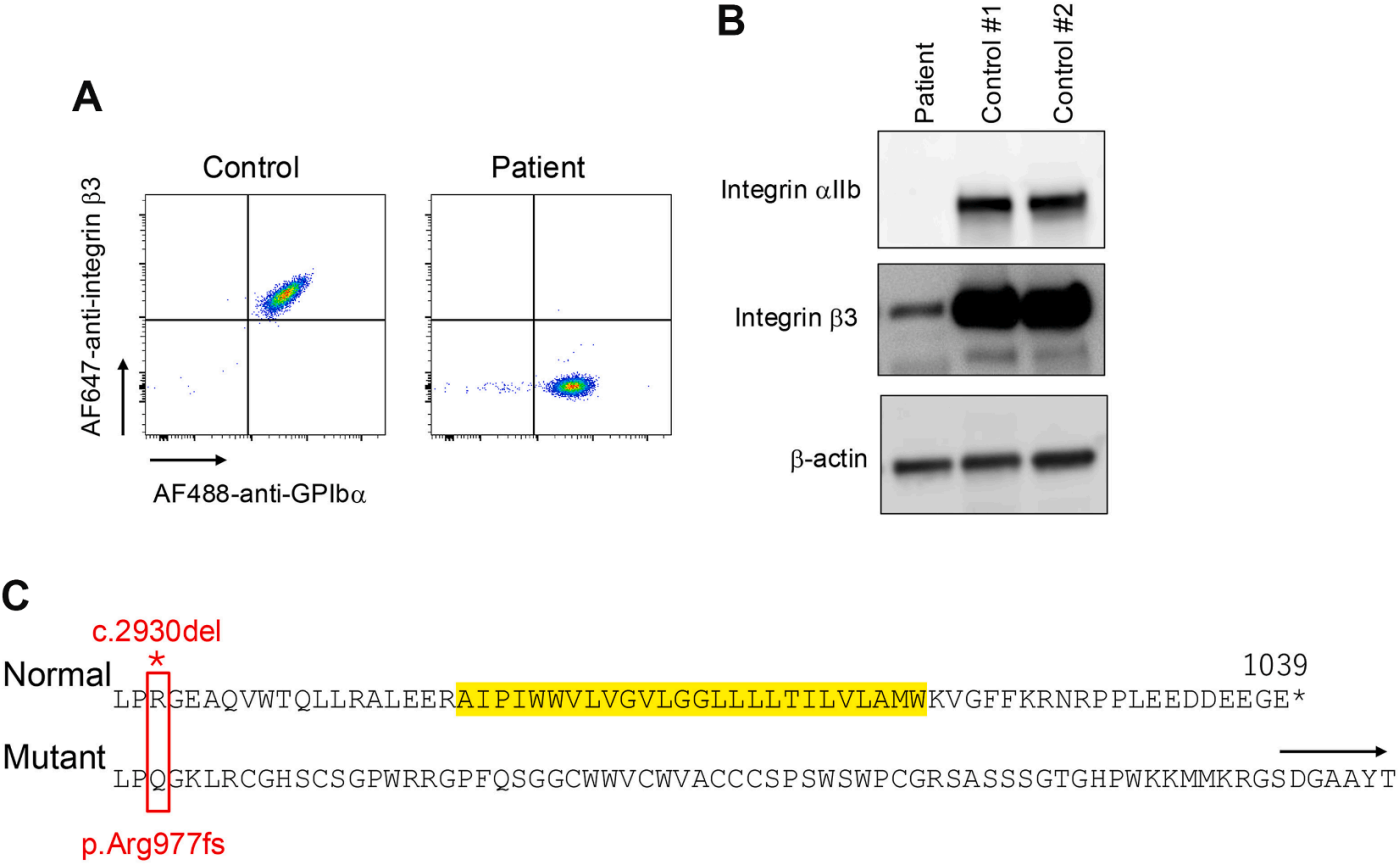
Defective expression of integrin α_IIb_β_3_ caused by a 1-base deletion in the *ITGA2B* gene. (A) Whole blood from the patient and a healthy control was stained with an Alexa Fluor 488 (AF488)-labeled antiglycoprotein Ibα antibody and an Alexa Fluor 647 (AF647)-labeled anti-integrin β_3_ antibody. Samples were analyzed using a FACSVerse flow cytometer (BD Biosciences), and data were processed with FlowJo software. Flow cytometric profiles of the platelet population are shown. (B) Washed platelets prepared from the patient and 2 healthy controls were lysed, subjected to sodium dodecyl sulfate–polyacrylamide gel electrophoresis, and analyzed by Western blotting using a rabbit polyclonal antibody against integrin α_IIb_, mouse monoclonal antibodies against integrin β_3_, and β-actin. (C) Alignment of the integrin α_IIb_ amino acid sequence in the region containing the 1-base deletion. The deletion causes a frameshift that alters the downstream amino acid sequence without introducing a premature termination codon. The transmembrane domain of integrin α_IIb_ is highlighted in yellow.

To confirm her platelet disorder, platelet protein expression was evaluated by flow cytometry and Western blotting. Both integrin α_IIb_ (CD41) and β_3_ (CD61) were severely reduced, consistent with type I GT (Figure 1A, B). Next-generation sequencing of *ITGA2B* and *ITGB3* identified a homozygous single-nucleotide deletion, c.2930del (p.Arg977fs), in *ITGA2B*. This mutation causes a frameshift without introducing a premature stop codon. However, the altered amino acid sequence disrupts the hydrophobic cluster that forms the transmembrane domain, resulting in the loss of α_IIb_ expression on the platelet surface even if the aberrant protein is synthesized (Figure 1C). No pathogenic variants were found in *ITGB3*. The trace amount of β_3_ protein detected by Western blot is not expressed on the platelet surface but is presumed to be retained intracellularly in the endoplasmic reticulum (ER) and/or Golgi apparatus and subsequently degraded.

**Figure 2:**
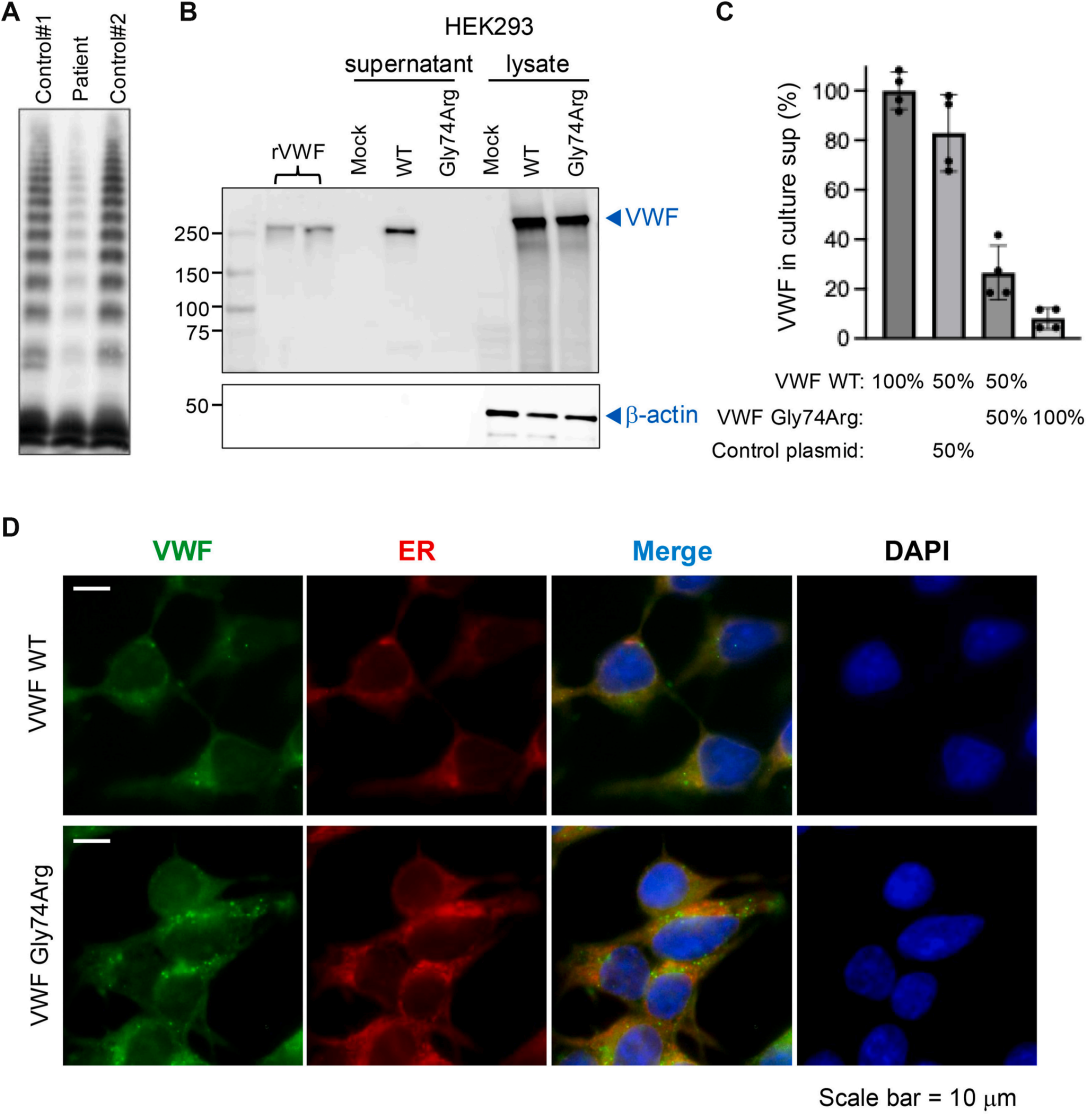
Effect of a propeptide missense mutation on von Willebrand factor (VWF) secretion. (A) Plasma VWF multimers were separated by electrophoresis on 1% SeaKem Gold agarose (Lonza) and visualized by Western blotting with an horseradish peroxidase-conjugated antihuman VWF polyclonal antibody (Agilent). (B) human embryonic kidney (HEK)293 cells were transiently transfected with plasmids encoding wild-type (WT) or Gly74Arg VWF. Intracellular and secreted VWF were analyzed by sodium dodecyl sulfate–polyacrylamide gel electrophoresis followed by Western blotting. Purified recombinant VWF (rVWF; 3.2 and 6.5 ng) was used as a control. (C) HEK293 cells were transfected with WT VWF, Gly74Arg VWF, or an irrelevant control plasmid expressing green fluorescent protein. VWF in culture supernatants was quantified by enzyme-linked immunosorbent assay using a rabbit antihuman VWF polyclonal antibody and expressed as a percentage relative to WT (set to 100%). (D) Immunofluorescence analysis of intracellular VWF localization. HEK293 cells transfected with WT or Gly74Arg VWF plasmids were stained for VWF and calreticulin (endoplasmic reticulum [ER] marker), and counterstained with 4′,6-diamidino-2-phenylindole (DAPI) to visualize nuclei. Images were captured using a BZ-X700 microscope (Keyence). Scale bars = 10 μm.

To explore the etiology of her concomitant VWD, we analyzed VWF multimeric status and the *VWF* gene. VWF multimer electrophoresis showed uniformly reduced band intensity, consistent with type 1 VWD (Figure 2A). Sanger sequencing of all exons and exon-intron junctions of the *VWF* gene identified a heterozygous missense variant (c.220G>A, p.Gly74Arg) in exon 3, located within the propeptide region and previously reported as a pathogenic mutation. Additional missense variants identified in this patient were p.Val471Ile, p.His484Arg, p.Gln852Arg, p.Thr1381Ala, and p.Val1565Leu. To investigate the molecular impact of the *VWF* c.220G>A (p.Gly74Arg) mutation, we introduced the variant into a VWF expression plasmid and transiently expressed it in HEK293 cells. Western blot of cell lysates showed intracellular synthesis of the mutant VWF comparable to that of wild-type VWF (Figure 2B). However, enzyme-linked immunosorbent assay of culture supernatants revealed markedly reduced secretion of mutant VWF (Figure 2C). Coexpression of wild-type and mutant constructs resulted in more than a 50% reduction in total VWF secretion compared with wild-type expression alone, suggesting a dominant-negative effect on VWF multimerization or secretion. Immunofluorescence microscopy showed that the mutant VWF was retained within the ER, colocalizing with ER markers, whereas wild-type VWF was efficiently secreted, with weak staining of secretory vesicles (Figure 2D). The *VWF* p.Gly74Arg variant lies within the propeptide region, which mediates VWF dimerization and multimer assembly in the ER. Our findings indicate that the p.Gly74Arg substitution in the VWF propeptide disrupts intracellular trafficking and secretion of the protein. The interaction between mutant VWF and wild-type VWF during dimerization in ER provides a plausible explanation for the dominant-negative effect on the secretion of wild-type VWF [7,8].

This case represents a rare example of coexisting GT and VWD, with distinct genetic lesions affecting platelet aggregation and plasma VWF secretion. Both mutations identified in this study have previously been reported as pathogenic variants associated with GT and VWD [9,10]. A particularly noteworthy aspect of our patient's clinical course was the development of severe gastrointestinal bleeding from intestinal angiodysplasia, which required surgical intervention. Gastrointestinal bleeding from angiodysplasia is a well-recognized complication of VWD, occurring most frequently in type 2A and type 3 VWD [11]. This pathophysiology is similar to that of acquired von Willebrand syndrome associated with aortic stenosis (Heyde’s syndrome)**,** in which increased shear stress promotes proteolysis of high-molecular-weight multimers, leading to gastrointestinal angiodysplasia and bleeding [12]. The functions of VWF in regulating endothelial integrity and suppressing abnormal vascular growth are considered underlying mechanisms [13]. In addition, cases with congenital platelet diseases, such as Bernard–Soulier syndrome and GT, have shown gastrointestinal bleeding due to angiodysplasia, though the mechanism is less well understood [14,15]. In the present case, although type 1 VWD is less commonly associated with angiodysplasia, its combination with platelet defects has produced a profound bleeding diathesis that could not be controlled by platelet transfusion and VWF replacement therapy.

The clinical implications of this finding are significant. In patients with congenital platelet disorders, persistent or disproportionate bleeding should prompt consideration of coexisting plasma coagulation or adhesive protein defects. VWD, being relatively common, may coexist with rare disorders, potentially compounding bleeding risk. The relative contribution of GT and VWF to bleeding severity is unclear at this moment. The patient’s father was affected by VWD but did not exhibit bleeding symptoms. However, his grandniece reportedly had VWD with bleeding manifestations, suggesting that VWD alone may contribute to the bleeding phenotype in this family. It is well recognized that bleeding symptoms in VWD can become more apparent in females because of hemostatic challenges associated with menstruation and childbirth, even among individuals carrying the same variant. The patient’s brother died in childhood from unexplained bleeding episodes, whereas her 3 sisters have not shown notable bleeding symptoms and have not undergone clinical or genetic evaluation. Unfortunately, blood samples from family members were not available for further genetic or laboratory analyses in the present study. Taken together, the family history suggests that VWD itself contributed to the bleeding tendency, while the coexistence of homozygous GT likely exacerbated the phenotype and resulted in the severe bleeding manifestations observed in the patient.

Our data highlight the importance of integrating molecular genetics and functional studies to understand atypical bleeding phenotypes. Genetic testing not only confirms clinical diagnosis but can uncover additional or modifying variants that influence disease severity. The combination of *ITGA2B* and *VWF* mutations in this case underscores the complexity of hereditary hemostatic disorders and the need for comprehensive evaluation, even when a primary diagnosis appears evident. Comprehensive genetic and functional assessment may facilitate accurate diagnosis and optimal management of complex bleeding disorders.
